# “We are the stakeholders with the most at stake”: scientific and autism community co-researchers reflect on their collaborative experience in the CONNECT project

**DOI:** 10.1186/s40900-020-00233-2

**Published:** 2020-09-27

**Authors:** Caroline Jose, Patricia George-Zwicker, Louise Tardif, Aaron Bouma, Darlene Pugsley, Luke Pugsley, Mathieu Bélanger, Jeffrey Gaudet, Marc Robichaud

**Affiliations:** 1 Maritime SPOR SUPPORT Unit, Université de Moncton, Moncton, NB Canada; 2 Research Laboratory on Chronic Disease Prevention, Centre de formation médicale du Nouveau-Brunswick, Moncton, NB Canada; 3 Department of Family Medicine, Université de Sherbrooke, Sherbrooke, QC Canada; 4 Autistics Aloud, Halifax, NS Canada; 5 Patient partner, CONNECT project, Maritime Strategy for Patient Oriented Research Support Unit, Université de Moncton, Moncton, NB Canada; 6 Vitalité Health Network, Centre hospitalier universitaire Dr Georges-L.-Dumont/Dr. Georges-L.-Dumont University Hospital Centre, Moncton, NB Canada

**Keywords:** Autism Spectrum disorder, Co-building, Co-learning, Multi-stakeholder, Patient engagement, Patient-oriented research, Patient partner

## Abstract

**Background:**

Little research describes the everyday challenges and needs of autistic adults. In order to fill this data gap, the CONtiNuity of carE and support for autistiC adulTs (CONNECT) project set out to learn about the health and well-being of autistic adults as well as their service and support needs. To do so, CONNECT welcomed autistic adults and caregivers of autistic adults as members of the research team, alongside researchers, policy-makers, service providers and health professionals. Autistic adults were involved in every stage of the research project and participated in team meetings held several times a year as well as in numerous email exchanges.

**Methods:**

Two feedback questionnaires were designed for this study: one for the scientific co-researchers and one for the autism community co-researchers (the project’s “patient partners”). Although the surveys varied from one another, they probed respondents to provide critical and constructive comments on issues that were central to their engagement in CONNECT. Four scientific co-researchers and four autism community co-researchers filled out the questionnaires. A comparative analysis was carried out on the responses provided to the open- and closed-ended survey questions as well as on complimentary data collected from the team’s documents.

**Results:**

CONNECT was seen as a positive experience for both groups. Highlights included: helping tailor and design research and its relevant materials to better suit the needs of the autistic community; establishing relationships and creating long-lasting friendships with other autistic adults; gaining a better understanding of the research process; and forging new connections with regional, national and international stakeholders. Areas for improvement include: establishing clear roles, responsibilities and expectations from the start; outlining a strategy to address unforeseen changes in project leadership; and creating a platform allowing for the involvement and participation of a more representative sample of adults on the autism spectrum.

**Conclusions:**

While not without its challenges, CONNECT demonstrates that a collaborative multi-stakeholder approach engaging autistic adults can be an effective model for conducting research on adult autism. Autistic adults and their caregivers can make the research process more open and accessible and make its outputs more relevant, useful and meaningful to the wider autistic adult community.

## Plain English summary

Autism Spectrum Disorder (ASD) is a lifelong condition. Yet, when compared to children on the autism spectrum, not much is known about the needs of autistic adults and the challenges and barriers that they face each day. Launched in 2017, the CONtiNuity of carE and support for autistiC adulTs (CONNECT) project set out to learn more about the everyday lives of autistic adults.

The CONNECT team included researchers, service providers, policy-makers, as well as autistic adults and caregivers of autistic adults. Early on, an autistic adult was promoted to the role of project co-lead. Autistic adults were involved in every stage of the research project, including co-producing this article. For this article, four scientific co-researchers and four autism community co-researchers (the project’s “patient partners”) filled out a questionnaire asking them to reflect on their experiences as members of CONNECT, to think about what they liked or did not like about the project and what lessons they learned from working together.

While not without its challenges, CONNECT was a positive experience for both groups. Including the voices and perspectives of autistic adults ensured that the study’s design, results and materials were more relevant and better adapted to the needs of the larger autistic community. Recommendations for future collaborative research initiatives of this nature include: having a contingency plan in place to respond to sudden, unforeseen changes in project leadership; providing autistic team members with key leadership roles; and establishing clearly defined roles, responsibilities and expectations for each team member.

## Background

In 2011, the Canadian Institutes of Health Research (CIHR) launched the national Strategy for Patient-Oriented Research (SPOR), a major new undertaking in the promotion, development and funding of participatory health research in Canada. Similar to initiatives launched in the United States [1, 2], the United Kingdom [3], Australia [4, 5] and elsewhere, SPOR views the lived experiences and perspectives of the public as an untapped skillset vital to conducting relevant and impactful health research. At the core of the SPOR philosophy are the terms “patient” and “patient engagement.” From the CIHR’s perspective, the term patient encompasses individuals with personal experience of a health issue or family members in their role as informal caregivers; in this sense, they are essential in identifying research topics and questions most relevant to the needs of the community they represent [6]. As for “patient engagement” (PE), the CIHR’s *Patient Engagement Framework* defines it as “meaningful and active collaboration in governance, priority setting, conducting research and knowledge translation.” [7] Although drawing on several decades of participatory research development, mainly in social science [8], the principles and values at the heart of SPOR have yet to permeate health research fully. While knowledgeable of the main tenets of patient-oriented research (POR), researchers perceive and identify many challenges in putting the approach into practice [9].

This reluctance to adopt participatory research approaches, including POR, has long plagued health research on people living with autism spectrum disorders (ASD) as well as other vulnerable and marginalized communities [10]. A lifelong neurodevelopmental condition, ASD manifests itself through a variety of social, sensory and communication differences as well as restricted, repetitive or inflexible behavior patterns [11]. Given the scientific rigors of the research process and the specificities of autism itself, it has been asserted that including autistic individuals as members of a research team is not feasible, nor practical, a viewpoint that has proven difficult to dispel [12]. There have been notable exceptions to this trend, however, and recent scholarship has highlighted the contributions of several pioneering and prolific autistic researchers, both in Canada [13, 14] and abroad [15–17], as well as ground-breaking network building and capacity building initiatives led and co-led by researchers on the autism spectrum [18–21]. These initiatives have allowed autistic researchers to reflect upon the relatively slow pace of change in autism research that has long conveyed “a discourse about us without us.” [20]

The CONtiNuity of carE and support for autistiC adulTs (CONNECT) project, launched in 2017, is another initiative that sought to challenge this long-standing position. It did so by using a POR-inspired approach to better understand the everyday needs and challenges of autistic adults, a segment of the autistic community that has long been overlooked by both researchers and policy-makers alike [22, 23]. More specifically, the goal of CONNECT was to shed light on the autistic adult community living in the three Canadian Maritime Provinces (New Brunswick, Nova Scotia and Prince Edward Island). There are one in 160 children on the autism spectrum worldwide [24]. In Canada, the prevalence of ASD among children and youth is one in 66 [25]. As ASD is a condition that occurs across the lifespan, the same prevalence can be expected in the adult population [26]. This means that an estimated 16,000 autistic adults reside in the three Canadian Maritime Provinces [27].

CONNECT brought together various stakeholders from across the region: researchers, service providers, policy-makers, health professionals as well as autistic adults and caregivers of autistic adults. CONNECT had four main objectives: 1) carry out a needs assessment survey to collect a wide range of health, socioeconomic and service needs data from autistic adults, caregivers of autistic adults and professionals working with autistic adults; 2) organize the region’s first-ever Autistic Adults Summit to bring together autism experts, community champions, autistic adults and caregivers of autistic adults; 3) organize a knowledge-sharing workshop to give stakeholders the opportunity to review and discuss preliminary survey results; and 4) develop knowledge-translation tools to help improve the planning, design and implementation of services and programs for autistic adults living in the Maritime Provinces. The data collected by the CONNECT survey has been the focus of several presentations [28] and conference posters [29], and a forthcoming scientific article will also be devoted to the analysis and interpretation of this data [30].

The present article will focus on the patient engagement component of CONNECT. For in addition to placing a strong emphasis on collaboration and co-learning, CONNECT was all the more innovative by the important place it gave to the voices and perspectives of autistic adults. Promoted as a research project *for* autistic adults *by* autistic adults, CONNECT was the first research project of its kind under the POR umbrella in Canada. Autistic adults were involved in all stages of the project, from the design stage to the knowledge translation stage, including co-producing this article. On average, the research team met several times a year, via in-person or teleconference meetings, and also maintained contact with patient partners through numerous emails.

While several studies on participatory research with autistic co-researchers have been published recently [16–19], few have outlined their engagement approach, context and outcomes in great detail. Reporting challenges and opportunities – both study-related and contextual – would contribute to identifying best engagement practices for autism research as well as reveal areas for improvement in POR’s semantic and core principles in research on neurodevelopmental conditions. As a result, the aims of this article are threefold: 1) to report on the context and methods of PE within the CONNECT project; 2) to document challenges involved in engaging members of the autism community in research and ways to overcome them; and 3) to illustrate, from the perspectives of both autistic and scientific co-researchers, the benefits that public collaboration brings to adult autism research.

## Methods

### Ethics approval

The CONNECT project was approved in 2017 by the Comité d’éthique de la recherche avec les êtres humains of the Université de Moncton, Moncton, New Brunswick, Canada (file no. 1617–068).

### Reporting guidelines

With the aim of contributing to the evidence base on patient and public involvement in research, and ensuring transparent and complete reporting of these study results, the long-form version of the Guidance for Reporting Involvement of Patients and the Public (GRIPP2) [31] was used to design and report this study (Additional file 1).

### Study participants

Study participants include four autistic adults and the caregiver of one of them, involved in the project since its early development (the project’s patient partners), and all four members of the research management team (the scientific lead, the project coordinator, a data analyst and a research professional) who actively worked on designing, implementing and supporting CONNECT’s participatory framework.

### Study design

During the results dissemination phase of the project, a feedback questionnaire was circulated among CONNECT’s patient partners (Supplemental Material 1). Modeled on existing qualitative PE assessment surveys [32], the questionnaire invited respondents to reflect upon various aspects of their involvement. Containing 13 questions and using mostly an open-ended format, the survey focused on issues such as recruitment methods, the nature, duration and scope of their engagement, whether their individual skills and lived experiences were sufficiently utilized, as well as the impact that their engagement had on the project and on their own personal development. The questionnaire also invited respondents to select the role that best described their engagement, proposing such titles as “advisor,” “leader” or “ambassador,” while leaving the door open for other perceived roles. The last question of the survey provided respondents with the opportunity to share any other additional comments or insights on the project as a whole.

At the same time, a feedback questionnaire was designed for and circulated among the research management team, whose members had worked closely on some or all of the components making up CONNECT’s PE framework (Supplemental Material 2). Their feedback questionnaire contained eight open-ended questions devoted to their experiences with PE in research, either in relation with, or previous to, CONNECT. The purpose of the questionnaire was twofold: first, to gather additional context with which to better frame and understand the related data; and second, to gather reflections and critical perspectives on CONNECT’s chosen PE approach, such as the challenges that arose from it and the impact it had on project outcomes.

### Sources of data

Four copies of the feedback questionnaire designed for the research management team were completed. As for the patient partners’ feedback questionnaires, four copies were also completed: three were filled out individually by self-reporting autistic adults, while the fourth was completed by the mother of an autistic adult. Her questionnaire contained the perspectives of two patient partners: her point of view as a caregiver and the point of view of her son who is non-verbal and has high support needs. Thus, the findings reported in this study are based on the responses of five patient partners.

Additional supporting data were retrieved from email correspondence, minutes of team meetings, social media content, media interviews, presentations made in various forums and settings, such as conferences and workshops, as well as from the scientific lead’s research journal. Finally, study participants provided input on and suggested edits to draft versions of this manuscript. This allowed to shed further contextual light on comments contained in the feedback questionnaires and on other data sources as well, thus providing for a more accurate interpretation of the self-reported perspectives.

### Analysis

The four completed patient partner feedback questionnaires reflect a variety of engagement strategies and scenarios. Because the patient partners perceived their individual roles within CONNECT differently one from another, we described their engagement experience, expectations, outcomes and impacts in relation to their roles.

Five main lines of enquiry guided the analysis of the patient partner feedback questionnaires: Expectations; Team Meetings and Engagement Support; Perceived Value of Engagement for the Autism Community; Personal Value of Engagement; and Satisfaction with Level of Engagement. In addition, before presenting the views and opinions expressed by the patient partners, several themes begin with a brief overview of the different measures and practices put in place by the scientific lead to facilitate PE. In the fifth and final theme outlined above, the patient partners provide an assessment as to the overall success of the CONNECT framework, and reflect on whether or not they would participate again in similar research projects in the future.

As for the feedback questionnaires completed by the research management team, they helped to delineate the context, history and nature of engagement in CONNECT. They also helped to guide the analysis of the perspectives voiced by the research management team regarding the challenges and impacts of PE, as well as the lessons to be learned from adopting such an approach.

Overall, this method helps to illustrate CONNECT’s approach to PE, as well as to highlight its strengths and weaknesses for those wishing to use this model in future research initiatives on adult autism research.

## Results

### Context of engagement in CONNECT

#### Engagement goals and scope: the scientific Lead’s viewpoint

The scientific lead was new to the field of adult autism research and wanted to compensate for this lack of familiarity by seeking “input and guidance” from members of the region’s autism community. While the scope of PE was “relatively clear,” the scientific lead was unsure about how to define patient partners’ engagement. Citing a lack of similar collaborative research initiatives on which to rely for guidance, the scientific lead pushed forward citing that “Too few examples [existed] when I started designing [the project in 2016] to follow some kind of guidelines on PE in autism research. So [I] just followed my intuition and adapted myself as we went on.” From the very beginning, welcoming autistic adults and caregivers of autistic adults as members of the research team was a foundational element of CONNECT and would remain so throughout the project’s lifespan. Consequently, choices about wording had to be made early on in the research process. This introduced a semantic hurdle in the early stages of the project as the autistic partners flagged the word “patient” as being an inadequate reflection of their lived reality:

Aware of the confusion surrounding “patient” in autism research, we introduced the term as relating, not to ASD itself, but rather to the health issues co-occurring with ASD, as these were a large focus of the CONNECT survey data. This was also consistent with CIHR’s definition of the term and agreed upon by autistic adults engaged in the team. We also strived to use other terms, like “partner engagement” instead of PE, as often as we could.

The autistic co-lead addressed this issue while speaking at an annual meeting of the Maritime SPOR SUPPORT Unit (MSSU). Invited to share her experiences as a patient partner, she explained that some words used in POR apply more readily to diseases like diabetes, but that the core values of POR fit any condition and can empower any community, so long as terms and concepts are clearly defined.

The intuitive nature of the CONNECT approach resulted in a “very flexible” PE framework, one that focused on collaboration as defined by the International Association of Public Participation’s (IAP2) *Spectrum of Public Participation* [33]. The ideas, suggestions and concerns of each team member were to be awarded equal value and given equal weight, while final decisions were to be the responsibility of the scientific lead. From the outset, she did not define or outline specific roles, proposing instead a flexible level of involvement reflecting each individual member’s needs, interests and availability. Although a consequence of her “lack of experience,” this PE framework, according to the scientific lead, “proved to work very well in [this] specific context,” and the path forward became more visible once project stakeholders began to acknowledge and appreciate the perspectives and contributions of the patient partners.

For the other members of the research management team, CONNECT also represented their very first foray into both PE and adult autism research. In addition, and similar to the patient partners, each research management team member joined CONNECT at different stages of the CONNECT project lifecycle. The data analyst was a member of CONNECT since the very start, having participated in the initial group meeting that officially launched the project. The analyst was convinced that having autistic adults as members of the team “was going to allow us to collect the ‘right kind’ of data, that is, hard data that would finally start to address issues and concerns at the very heart of that community.” As for the project coordinator, she came aboard shortly before the Autistic Adults Summit and would play a vital role in planning and organizing this important event, as well as all future project activities. Although she had wondered “whether or not I really had the necessary training to adequately support the autistic patient partners and their needs,” the project coordinator was convinced that engaging with autistic adults in research “was definitely going to make CONNECT more pertinent to members of that community and raise more awareness about their hopes and their struggles to gain acceptance.” The research professional joined the CONNECT team during the knowledge-translation stage, and was the last member to join the research management team. He was struck by the level of comradery that existed at all levels of the project, and learned about the history of CONNECT and the nature and scope of the contributions made by the autistic adults: “My goal, at that point, was simply to help keep things moving forward, building on the team’s past successes, and to continue to make CONNECT a space where patient partners felt welcomed and valued.” While their responses to the feedback questionnaire also highlight the more spontaneous or instinctual nature of the project, the research management team, who are all members of the MSSU, all shared the feeling of contributing to a groundbreaking research initiative whose outcomes were going to have, as the data analyst stated, a “direct, positive and long-lasting impact” on the region’s autistic community.

#### History and nature of engagement

Patient partners had various degrees of involvement in CONNECT, with some choosing to be involved on a punctual basis, contributing their time, insights and perspectives at key moments in the project lifecycle whereas others had a longitudinal involvement. Some were recruited early on in the project and participated in the inaugural CONNECT planning meeting, while others joined the research team after the project had been launched. The patient partner feedback questionnaire reflected this flexible and open approach towards PE.

Several autism co-researchers were recruited directly by the scientific lead. The future project co-lead was invited to participate in CONNECT after speaking at an autism conference in Nova Scotia. The scientific lead, who was in attendance, saw an opportunity to introduce herself, talk about the new project on adult autism and explain her desire of including the voices and perspectives of autistic adults. Early on, the newly recruited autistic partner was concerned about how the project was developing. From her perspective, the project was not going to attain the desired level of participation among its target population since it was not sufficiently adapted to the everyday realities of autistic adults. She argued that more had to be done to ensure appropriateness of the research question, as well as that of the phraseology, terminology and vocabulary used in the survey and in all other relevant project materials. To preserve the newly established partnership, and to recognize the numerous and important contributions of the autistic adult partners, the scientific lead suggested that the autistic partner assume the role of co-lead. This decision would appropriately recognize her role, help CONNECT obtain a higher survey participation rate from within the region’s autistic community, and help produce results more relevant for autistic adults and their caregivers.

The caregiver, who was also involved from the very early stages, was also recruited directly by the scientific lead. As a patient partner, the caregiver helped to design and draft the needs assessment survey and contributed to the development of a strategy for both disseminating the survey and recruiting participants. As for her son, his real engagement in CONNECT began during the knowledge-translation stage with the production of a video highlighting some of the key findings of the survey, a video in which the autistic adults themselves were to be the main stars.

Two autistic self-advocates joined the team during the Autistic Adults Summit held later that same year. One was directly recruited by the co-lead, whom she described as “one of the first Autistic person [s] that I connected with online,” a response that highlights the importance of the Internet as a communication tool for autistic adults seeking to establish connections with peers near and far [18]. The new autistic partner, who described her role as that of “patient advisor,” then turned to her own network of friends to promote the upcoming summit and to secure the participation of autistic adults for one of the event’s panel sessions. In this way, she was able to recruit the second autistic self-advocate who would eventually come to see his role as that of both “advocate” and “ambassador.” As before, no specific roles were assigned and each autistic self-advocate had the freedom to decide their own level of engagement.

Engagement in CONNECT took on multiple forms. Patient partners and the research management team worked collaboratively to develop a recruitment strategy to promote the needs assessment survey within the larger autistic community. In so doing, the autistic partners became effective spokespersons for the project, sharing information about the goal and objectives of the project in their own words. They did so by way of newspaper, radio and television interviews, social media posts, and a video that garnered the attention of both local and national media. Patient partners were consulted for suggestions on the type of accommodations needed to make project-related events and activities more autism-friendly and, thus, more accessible. They presented project results at various regional [28], national [34] and international conferences [29, 35] where they also discussed CONNECT’s implications for future PE initiatives. Patient partners participated in preparing meeting agendas and in revising the minutes of the meetings. They were also important architects of various knowledge-transfer tools, including scientific article manuscripts, conference posters [29, 34, 35], newsletters and videos. Positioning herself as a knowledge broker, the scientific lead saw that each autistic partner had equal decision-making power on all aspects of the research.

### Perspectives of autism community co-researchers

#### Expectations

The autistic co-lead understood the nature of her engagement, describing it as participation “in an inaugural meeting that was aiming to locate the gaps for autistic adults in the Maritime Provinces.” However, the scope of her involvement was, from the outset, not so clear. In terms of initial expectations, the autistic co-lead emphasized the data collection component of CONNECT, underlining that the project was going to provide “a real look” into the region’s autistic community and into their “unique needs and wants,” timely data for autistic adults and decision-makers alike. While those expectations had been met, her hope was that the project would identify ways to bridge those gaps. Reflecting more broadly on the extent of experiential knowledge within the research team itself, the autistic co-lead considered it to be low at the beginning, which explained her sentiment of feeling “a bit outnumbered.” This feeling eventually subsided as more autistic adults got involved in the project.

The caregiver’s experience was similar to that of the autistic co-lead since the scope of her involvement also lacked clarity from the start. This lack of direction was compounded by an apparent lack of dialogue and communication within the team itself; for example, she noted that “there was committee work done that I wasn’t aware of until afterwards.” In terms of expectations, the caregiver was hoping that the project would provide more space and give more importance to “issues affecting people who share [my son’s] diagnosis.” As mentioned previously, the research management team acknowledged its lack of practical and theoretical experience in PE when launching CONNECT, and the scientific lead in particular “was not sure how to get it right” once the project got off the ground. In addition, the research management team sometimes received comments from patient partners who felt that they were not sufficiently included in the project. In its efforts to accommodate them, however, the team unintentionally excluded others. This proved to be a major challenge for CONNECT, and demonstrated the need to take early action in order to create a truly open, welcoming and inclusive space for everyone on the autism spectrum.

As for the two autistic self-advocates, their expectations were similar to those of the co-lead, in that they both hoped to obtain useful data describing the everyday lives of autistic adults in the region, expectations that had been met. The “patient advisor” expected a high response rate for the survey, but acknowledged that this “seems to have been a challenge,” echoing statements made by the co-lead regarding her own unsuccessful attempts to recruit more survey participants.

#### Team meetings and engagement support

Several initiatives were put in place to support, encourage and sustain patient partner engagement. The scientific lead strove to create a work environment that was conducive to open dialogue and to the frank and respectful exchange of opinions and ideas. Another potential barrier to PE was removed by pre-paying or reimbursing autistic adults for all travel, meal and lodging-related expenses, thus permitting them to attend some team meetings in person and to participate in other project-related activities and events. On the issue of compensation, there were no published guidelines available when CONNECT was launched. This situation would last until the very final stages of the project since it was only in the Spring 2020 that a *Patient Partner Compensation and Reimbursement Policy* was adopted by the MSSU, in part as a result of the CONNECT experience [36]. Without such a policy, it had been agreed at the outset that patient partners would participate as volunteers and that all of their CONNECT project-related expenses would be reimbursed.

Project meetings were held occasionally in local autism resource centres, providing autistic partners with an environment that was familiar and already adapted to their particular needs. Agendas were circulated for feedback before meetings (mostly held via teleconference), doodle polls were conducted to choose a meeting time convenient to the majority of team members, and minutes were circulated following meetings for review and final approval.

The autistic co-lead noted that the research management team had provided support in the form of detailed emails and explanations regarding the specific objectives of the meeting and related materials. In general, she felt that she had enough time to “take everything in,” to provide feedback, and to request additions to the meeting’s agenda.

The caregiver thought that meetings could have been more useful for those attending. “Clarity of questions/roles/work outcomes and by whom would have helped,” she wrote, reiterating the importance of having roles and responsibilities clearly defined. The issue surrounding lack of representativeness was also noted, with the caregiver feeling somewhat excluded during meetings because discussion topics “started with presumptions that weren’t compatible with our family’s experience.” The caregiver observed similar issues during filming of the CONNECT video: “Questions like ‘what does equality mean to you’ take a great deal of time to process and to set up situations where an answer might be possible.” Although grateful for the open and considerate approach that was used to recruit her son for the video, the caregiver felt that more care should have been taken to make the concepts and ideas at the heart of the script and scenario more accessible and relatable to her son.

The autistic self-advocates both appreciated the collaborative nature of the work and the support of the research management team that allowed for time, patience and courtesy to be central values in the way the meetings were conducted. They did feel overwhelmed, however, by the quantity of information that was disseminated in the lead-up to certain major activities or events, especially those involving significant travel. While appreciative of the team’s efforts in handling the logistics of travel and the production of useful travel itineraries, they suggested that the research management team do a better job in selecting information that could be more easily communicated verbally. This would help avoid an overabundance of very long and detailed emails and attachments, exchanges that made their preparation more difficult and stressful.

#### Perceived value of engagement for the autism community

The autistic co-lead felt that her engagement was “invaluable” for CONNECT as she had several years of experience as an autistic advocate and a lot of experiential knowledge. Her perspectives and insights were all the more significant since, as she herself acknowledged, “Autistic adults rarely get to be as involved as I was in research projects about Autism.”

Patient partners welcomed the fact that CONNECT included the perspectives and voices of autistic adults, an approach described by one autistic self-advocate as “vital” and long overdue in autism research. Another autistic self-advocate echoed these sentiments, writing that she was “exceedingly pleased” to witness research on autism being co-led by a member of the autistic community, and that “[i] nclusion leads to good science [and] to more relevant research findings.” During one particular meeting, she led the team in an empathy-mapping and persona-creation workshop that offered new insights into the hopes, dreams and fears of autistic adults and their families while, at the same time, providing new and rich qualitative data to both complement and enhance the quantitative data collected through the needs assessment survey [37, 38].

Patient partners also enlightened the research management team as to the negatively-charged meanings hidden behind seemingly innocuous terms, notably “patient,” a word commonly used within the POR community but viewed as ill adapted to a project on adult autism. As the autistic co-lead explained: “Some words I understand are still very much works in progress – but autism is most definitely not a disease. It’s important to move away from that language in particular as it creates a cure mindset.” Such interventions demonstrate how engaging members of marginalized communities can help broaden one’s horizons and educate others regarding stereotypes and misperceptions, notably by “inform [ing] the group about potential offensive or unclear language or assumptions.” [18] In choosing “patient” as the cornerstone of its national participatory research strategy, the CIHR acknowledged that the term was far from universally accepted. In its *Patient Engagement Framework*, the federal funding agency recognized that the word “may initially evoke a range of meanings or limitations depending on the audience.” [7] Nevertheless, the CIHR meant for the terminology to be “inclusive.” [7] As mentioned earlier, the team was sensitive to this and sought to demystify the term “patient” and to contextualize its use within the POR framework. By stressing the value and importance of including the perspectives of autistic adults in research, the autistic self-advocates agreed with the co-lead who maintained nonetheless that POR- and PE-inspired initiatives “will provide better outcomes for us all.”

On the question of engagement impact, the caregiver explained that her son’s involvement in CONNECT had led to greater public awareness of and sensitivity to the specific issues facing autistic adults with high support needs:

[My son’s] brother decided he’d like to speak out about services for Autistic adults through a persuasive writing essay at [his university], which his professor asked to publish [39]. It’s been shared thousands of times and was a starting point for some good family conversation [ … ]. Dozens of people in [my son’s] circle including staff at two special care homes have become more involved with capacity/competence building and more understanding of the importance of choices and of understanding subtleties of atypical communication.

Even though it occurred much later in the project lifecycle, her son’s involvement did have a noticeable and lasting effect on his immediate surroundings.

#### Personal value of engagement

CONNECT had an important impact on the patient partners’ personal development. “My world has really opened up,” wrote the autistic co-lead in her feedback questionnaire. She developed friendships with other autistic adults, which provided her with a much-needed “support system.” She met other people who were just like her and who, on a daily basis, faced similar challenges and barriers. She also became more aware of and sensitive to the realities of autistic adults with more complex support needs and to the specific challenges that they and their families face every day. In one of her many public speaking engagements on CONNECT, the co-lead described the two-day filming session for the video as the “best weekend ever.” Together, these unexpected outcomes of the CONNECT project were so life affirming and invigorating that the co-lead described them as a form of “Autistic Oxygen.”

The other patient partners also referenced the transformative impact that their participation in CONNECT had on themselves, on the research team, and on the wider community. The two autistic self-advocates saw themselves reflected in the study results and, like the co-lead, reported having developed new friendships, established new connections, recruited new allies and obtained greater insights into the research process. The Autistic Adults Summit had played a defining role in this, as one the autistic self-advocates explained in a blog post: “These adult autistics became my good friends and we all became very close over the 36 hours we were [ …] together. We shared stories that were similar in nature and context when dealing with the neurotypical world we all live in.” [40] Participation in regional, national and international conferences also provided patient partners with new and extensive networking opportunities, making connections not only with other autistic adults, but also with researchers, autism advocates and community leaders both from home and abroad; on one occasion, the latter described CONNECT’s patient partners as “awesome advocates” [41].

#### Satisfaction with level of engagement

The autistic co-lead felt left out of data interpretation and explained that she would have needed a better understanding of how CONNECT survey data were interpreted. While interpretation of the survey results was carried out collectively, during in-person team meetings, the data themselves were analyzed prior to the meetings and were presented in tables of results. In terms of her own contributions to the project, the co-lead felt somewhat disappointed in two key areas: “I wish I could have been more helpful in planning the Summit [and] I wish I could have gotten more Autistics to fill out the survey.” However, it was not specified whether or not these shortcomings stemmed from a lack of opportunity provided by the research management team.

Lack of inclusion and representativeness were issues at the heart of the caregiver’s questionnaire. Missing from the project was “more involvement of those able to share the perspective of adults with severe communication challenges and those with very high support needs.” This element constituted a caveat for CONNECT, one that needed to be highlighted when presenting results and when discussing the project’s PE framework. In other words, for the caregiver, explaining “whose voices were not included (and why)” needed to be a core element in any discussion of CONNECT’s results. Nevertheless, the filming of the CONNECT video proved to be a positive and fulfilling experience for her son: “[He] visibly enjoyed spending time with people who had an interest in connecting with him.”

The two autistic self-advocates felt that the nature and scope of their engagement were well defined early on and that they were provided with ample time and opportunities to provide input, appreciating the equal voices given to all autistics involved. Both the scientific and autistic co-leads played an important role in making the new autistic partners feel welcome and in helping them better understand CONNECT’s collaborative philosophy. As one of the autistic self-advocates explained, the two co-leads both outlined:

… the scope of the project, how the input of Autistic individuals and others shaped the research questions, how the various stakeholders were presented the results and had an opportunity to provide perspectives on the results, and how the next steps were discussed in continued involvement.

One of the autistic self-advocates mentioned feeling “at home in that sense of belonging with [his] peers,” reiterating the co-lead’s sentiment of being more at ease once additional autistic adults joined the team.

Shortly after filming the CONNECT video in early 2019, the scientific lead was forced to suddenly withdraw from the project due to ill health. In the days and weeks that followed, the autistic co-lead sensed that the research management team was having difficulty adapting to this new reality and that the project was losing its way, a sentiment echoed by the other patient partners. From the autistic co-lead’s point of view, there were many lessons to be learned from this unfortunate experience, one that left her with some emotional scars. In particular, she pointed to the need of having a contingency plan in place from the very beginning in order to deal with the sudden absence of a key team member, especially one with whom the core relationship of trust had been established and whose energy and vision had been the principal guiding force for all involved. After some time and adjustments, the group dynamic and team synergy were eventually reestablished. In the final edition of the CONNECT newsletter, the autistic co-lead reflected on how the project had been a learning experience for all involved, herself included: “It’s not all gone smoothly, but one of the most important things this process has taught me is how vital learning from those mistakes is.”

When asked if they would participate again in similar research initiatives, all five patient partners answered in the affirmative. Speaking on behalf of her son, the caregiver answered that he “found it a pleasant experience.” The autistic co-lead has since accepted a role as Patient Partner within the MSSU, an example of her continued engagement in POR. This position enables her to share her knowledge, experiences and insights with staff at all levels of the organization and with the wider SPOR community in an effort to better promote POR and to improve the overall PE experience. The autistic self-advocates were also open to reengaging with similar research initiatives in the future and have since done so. When the MSSU organized a stakeholder engagement meeting in New Brunswick in early 2020 to inform its new business plan, the two autistic self-advocates participated in the event and offered their perspectives and insights alongside those of clinicians, administrators, policy-makers, universities, health care organizations and MSSU staff, to name only a few. Moreover, both autistic self-advocates are currently involved in new POR-inspired research initiatives in the field of adult autism research.

### Perspectives of scientific co-researchers

#### Expectations

The scientific lead’s expectations were met in several ways. By inviting members of the autism community to join the research team, she was able to “design and conduct a meaningful and relevant research project,” despite her own lack of expertise in autism research. As the project and its PE component developed over time, the scientific lead also received increasing support from fellow researchers with proven track records in autism research. This, in turn, reduced her fear of “not being taken seriously,” increased her own level of self-confidence, and demonstrated the project’s overall trustworthiness in terms of design, methods and outcomes. Considered to be a liability at first, CONNECT’s intuitive and flexible approach to PE proved to be, in the end, one of the project’s great assets: “the uncertainty around the needed level of engagement, the lack of clear guidelines for patients and researchers and the lack of confidence in PE from all involved [ …] were the key elements that enabled building trust, co-learning, companionship, and adaptability.” For the scientific lead, this was one of the many unanticipated results of the project, alongside what she described as the creation of a “Neurosiblings Family,” a development highlighted by the autistic co-researchers themselves.

Describing herself as a promoter of the POR philosophy in the Maritime region, the scientific lead also wanted to demonstrate that PE was achievable in all manner of health research. In this regard, CONNECT proved to be a field of riches:

[The project provided] tools and proofs of concept for further use of PE in health research [ … ] the development of a PE tool kit [ … ], various opportunities for sharing impact and outcomes of PE in CONNECT through different media, and numerous training improvements in PE at a regional and national level.

As for the other members of the research management team, they all agreed that CONNECT had been a rewarding, “eye-opening” and enlightening experience, both on a professional and personal level.

#### Lessons learned

CONNECT was not without its growing pains and the research management team readily acknowledged that the learning curves were at times quite steep and that there had been occasional misunderstandings. The scientific lead and the data analyst both recognized that certain teachable moments had been overlooked. Although they tried to explain the analytical processes that lay behind the creation of results tables, they admitted that more time and effort could have been devoted to clarifying, in lay language, the processes involved in transforming large datasets into comprehensive results tables. Managing expectations was another key lesson learned: members of the research management team realized the importance of accurately assessing time and resource requirements when planning key deliverables as a way of avoiding frustrations and reducing the risk of disappointment among and disengagement on the part of patient partners.

Echoing the autistic co-lead, the scientific lead also recognized the need to draw up an “alternative leadership plan” and spell out the project’s core values from the beginning. This could help steer the team in the right direction, especially “in [times] of emergencies like [the one] we had when I had to leave the project for serious personal health issues.” As for assembling a team of patient partners that truly reflected the myriad faces and voices of autistic adults, this proved to be an elusive goal:

More care should be taken to make sure that when we say we are inclusive and reflect the perspectives and lives of Autistic adults, we do provide a platform adapted to include them all. I failed in having equal representation of non-verbal or more disabled Autistics and they were outnumbered by self-advocates.

Thus, on the issue of inclusiveness and representativeness, there was a great deal of commonality between the scientific lead’s feedback questionnaire and that of the caregiver.

Further, the scientific lead pondered on an ethical by-product of engaging autistic adults in participatory research. When nearing the final phases of the project, she reflected over the long road that the team had travelled together, realizing that the close-knit bonds established and nurtured between CONNECT’s scientific and autistic co-researchers now represented somewhat of a double-edged sword:

There is a real ethical dilemma in being emotionally and personally engaged, both for the researchers and the community members. How can we make sure that PE gets as far away as possible from tokenism when we only have several months or a couple of years to develop a trusting relationship that may end abruptly when the project ends. This has the potential to leave a gap in the lives of volunteers. Research on this topic is required to inform best practices in the final stages of POR.

While such reflections may be breaking new ground in the field of POR, recent studies have begun to explore the issue of “muddled relationships” within other models of collaborative research partnerships and to examine the issues that these relationships pose to both members of the community and researchers alike [42]. Lessons learned from the CONNECT experience suggest that a POR approach has a potential to create “relationships that blur the line between friendship and a formal research relationship” [43].

As mentioned previously, open and frank dialogue were among CONNECT’s core values. This meant that patient partners were always in a position to identify areas needing improvement and to bring them to the attention of the research management team so that the issues could be addressed and resolved. Table 1 highlights some of the key lessons learned through the input and constructive feedback received from the patient partners. It summarizes the contents of both categories of feedback questionnaires as well as ongoing discussions between the research management team and the autistic co-researchers.

**Table 1:** Some Key Take-Aways from the CONNECT Experience with Patient Engagement

Communications	Maintain frequent contact with patient partners
	Enquire on mode of communication preference
	Keep emails brief
	Avoid multiple subjects or discussion topics in the same email
Roles and Responsibilities	Provide patient partners with meaningful opportunities to engage
	Consider providing patient partners with key leadership roles
	Identify clear roles and responsibilities for all team members
Project Planning	Draft the project’s core value statements as well as a contingency plan to deal will unexpected changes in project leadership
	Manage team expectations by assessing time and resources needed to produce key deliverables
	Provide adequate support to allow for full participation in project-related activities and events
	Provide reimbursement to patient partners for out-of-pocket expenses incurred from their involvement

## Discussion

It is well known that the research community tends to resist the idea that the balance of power in terms of key scientific decision-making processes is to be shared equally between researchers and members of the autistic community. Research priorities, funding decisions, study design and implementation, interpretation and dissemination of research findings, all of these components are said to constitute the “exclusionary domains” [44] of academics. The autistic co-lead addressed this issue directly in her feedback questionnaire. While fascinated “to be this up [and] close” to the research process, she recognized that this was very much a departure from the norm. Similar to other collaborative research initiatives involving autistic adults as co-researchers, CONNECT has clearly shown that “[a]utism’s many advantages are not part of the diagnostic criteria,” [45] and that insights from autistic adults are a good reference point for other teams wishing to conduct collaborative research on adult autism. As one autistic partner frequently reminded the research management team: “We are the stakeholders with the most at stake.”

CONNECT demonstrates that clear, well-delineated expectations and clearly defined roles for each team member are required from the very outset in order to fully engage and maintain trust with the autistic community. As the autistic co-lead and the family caregiver both pointed out in their feedback questionnaires, the scope of their own engagement was rather vague from the very moment they joined CONNECT. As mentioned in the “Context of Engagement” section of this article, the scientific lead was confronted early on by a lack of concrete examples on how to effectively conduct POR-inspired research projects involving and engaging members of the autistic community. Thus, from the project-design phase onwards, she relied on her instincts, revisiting, adjusting and adapting her approach as time went on and as specific issues arose and required attention.

CONNECT is the latest in a series of initiatives that have helped to foster “a burgeoning, merged community of research practice.” [46] In other words, by using a POR-inspired approach, CONNECT provided one answer to the often-posed question: “What does good public involvement [in research] look like?” [47] As the number of research initiatives including autism community co-researchers increase, so too have the number of guidelines and frameworks that seek to provide a useful and evidence-based road map to those researchers wishing or planning to undertake such a journey [48, 49]. Many of the lessons learned from CONNECT, such as the need for inclusiveness, transparency, clearly defined roles, responsibilities and expectations, effective and efficient communication tools and strategies, echo these emerging best practices in the field of participatory research involving autistic adults [49]. But CONNECT also shows that such frameworks must also include contingency measures aimed at mitigating the potential risk caused by the sudden absence of a key team member. That way, research momentum will not slow down, the project will not be left directionless, and best practices will not be partially or fully abandoned.

To conduct a collaborative research project with members of the autism community, transparency is key. The research process needs to be explained to and demystified for non-academic team members so that they may better understand all of its phases and components, latency periods, and time and financial constraints, including whether or not patient partners will be reimbursed for out-of-pocket expenses. Preliminary results of the CONNECT needs assessment survey showed that the median annual income of autistic adults in the Maritime Provinces is well below the Canadian poverty line [28]. Such data served to highlight even more the importance of reimbursing project-related expenses as a means of supporting PE and removing barriers to involvement. With a new *Patient Partner Compensation and Reimbursement Policy* in place at the MSSU, the funding proposal for an eventual phase II of CONNECT would subscribe to these guidelines. In terms of roles and responsibilities, it is important to let patient partners choose the title that resonates the most with them and which allows them to create a sense of belonging as members of the research team.

While many strategies exist for identifying and recruiting patient partners [50], CONNECT illustrates the usefulness and efficacy of personal contacts and of one-to-one relationships and discussions in promoting the objectives, values and benefits of POR. This is especially the case with autistic adults who are often marginalized and isolated, a reality that continues to make outreach one of the main barriers to conducting research on and with this segment of the autistic community [51–53]. That some of CONNECT’s autism co-researchers mentioned their disappointment in not achieving a high survey participation rate testifies to the challenges that persist when reaching out to autistic adults. CONNECT shows that these hurdles are not insurmountable.

The autism community has identified certain key priorities regarding autism research, notably that the latter should focus more on finding concrete ways of improving the daily lives of both autistic persons as well as members of their immediate and larger support networks [22]. Through its POR approach, CONNECT focused on issues relevant to the autistic adult community. As a result, the project has helped increase the hope and trust that the latter has in both research and policy. By highlighting the key leadership role given to an autistic adult and by allowing ample time and space for autistic adults to share their own personal experiences with autism, the Autistic Adults Summit led to a greater recruitment of autistic partners and to an increase in survey participants. Some autistic advocates questioned the Summit’s scope and choice of presenters and panelists, considered to be, in both cases, much too narrow [54]. Such criticisms demonstrate the challenges in truly representing the different voices and perspectives making up the autistic adult community, an issue at the very heart of the caregiver’s feedback questionnaire. CONNECT did not resolve the tensions that exist within the autistic community resulting from the large variety of autistic traits as well as autism-associated health issues [22, 40]. Yet the project did help foster a better appreciation and understanding of the source of those tensions. The autistic co-lead herself acknowledged that her participation had increased her awareness of and sensitivity to this very issue.

While it constitutes “one of the most common barriers” to engaging and involving patients as research partners [55], the issue of training was not raised by patient partners in their feedback questionnaires. It is worth noting, however, that the CIHR has developed an introductory workshop on the principles and values of POR called “Foundations in Patient-Oriented Research,” which, ideally, is to be co-delivered with the help of a patient partner [56]. Over the life of the CONNECT project, all members of the research management team received this training and two are certified to offer the training. It may be useful to provide such training in the future to patient partners, especially if there is a phase II of CONNECT.

POR cannot truly flourish if patient partners feel unappreciated, undervalued or ignored [57, 58]. Researchers must recognize that patient partners can bring clear added value to a project and, as a result, need to create an atmosphere which truly encourages and supports their involvement and engagement. With CONNECT, the participation of autism co-researchers was far from tokenistic, although some additional accommodations would have allowed for greater participation on the part of some patient partners, especially those with high support needs. On the whole, the insights of the patient partners proved invaluable in developing knowledge-transfer tools adapted to the needs of the autistic community as well as in organizing autistic-friendly meetings, activities and events. By creating a welcoming environment and recognizing the specific expertise that each project partner brought to the table, the team also benefited from particularly formative and enlightening exercises, notably the empathy-mapping exercise that was proposed and facilitated by one of the autistic self-advocates. CONNECT shows that members of the autistic community constitute the “de facto experts” [59] on issues regarding the everyday needs of people on the autism spectrum. To help foster a health research culture more open to the principles and values at the heart of POR, the scientific lead developed a PE orientation tool kit to help researchers better plan for the involvement of patient partners from the outset and to help them maintain this engagement – or what she called a “sense of belonging” – throughout the project lifecycle, notably by regularly revisiting the expectations of team members. By adopting an approach founded on co-learning, co-building, respect and inclusiveness, CONNECT revealed POR’s great potential to affect positive change for members of vulnerable and marginalized communities.

## Conclusions

By incorporating and listening to the authentic voices and unique perspectives of autistic adults and caregivers of autistic adults, CONNECT has shown that SPOR has the potential to make adult autism research more impactful and more relevant to members of that community. CONNECT reduced some of the main barriers to adult autism research, including autistic adults’ lack of trust in research, ill-adapted outreach and recruitment strategies, and common public and researcher prejudices on autism. The project was not without its challenges and both the patient partners and the research management team identified several key areas in need of improvement, reassessment and reexamination. Future collaborative research initiatives need to adopt more flexible and inclusive practices so that adults from across the autism spectrum can be truly involved and engaged in research, and have their voices and perspectives heard [60]. Nevertheless, in remaining faithful to POR’s core principles, the CONNECT model proved its potential to make research more accessible and more relevant to members of the autistic adult community. All members of the research management team and many of the patient partners have expressed an interest in initiating a second phase of CONNECT, if financial conditions will allow it, and many of CONNECT’s patient partners are also involved and engaged in other POR-related research initiatives.

CONNECT also reveals some of the unanticipated results and transformative effects of PE. When the name “CONNECT” was initially chosen for the project, it was meant to communicate a paradigm shift in autism research by initiating a dialogue between autistic adults, service providers and policy-makers. When autistic youth reach adulthood, they often get “disconnected” from the various supports and services that were available to them during childhood. Families of autistic adults invariably described this experience as “falling off a cliff” [61] or as entering a “complete void.” [62] However, as time went on, the name of the project took on a completely new meaning, bringing into clearer focus the various human and personal “connections” that were forged among autistic adults themselves, life-changing relationships that continue to flourish to this day. A greater sense of personal empowerment and belonging are just some of the positive outcomes of patient-centered research [57]. On the whole, CONNECT helped break down social barriers and reduce the geographical distance that has long separated autistic adults from their peers. As for the researchers themselves, CONNECT allowed them to do research in a more humanistic way, placing empathy and understanding at the core of the process. While all partners benefited from their participation in this collaborative research venture, it is undoubtedly society as a whole who will gain the most from the acceptance and inclusion of people on the autism spectrum, whose skills, abilities and lived experiences are to be acknowledged and valued.

## Supplementary information

**Additional file 1.**

**Additional file 2.**

## Data Availability

Data sharing is not applicable to this article as no datasets were generated or analyzed during the current study.

## Abbreviations

CIHR: Canadian Institutes of Health Research
SPOR: Strategy for Patient-Oriented Research
PE: Patient engagement
POR: Patient-Oriented Research
ASD: Autism Spectrum Disorder
CONNECT: CONtiNuity of carE and support for Autistic adulTs
GRIPP2: Guidance for Reporting Involvement of Patients and the Public
MSSU: Maritime SPOR SUPPORT Unit
SUPPORT: Support for People and Patient-Oriented Research and Trials
IAP2: International Association of Public Participation
