# Supplementary material for: “We are the stakeholders with the most at stake”: scientific and autism community co-researchers reflect on their collaborative experience in the CONNECT project
Source: Res Involv Engagem. 2020 Sep 27;6:58. doi: 10.1186/s40900-020-00233-2 (PMC7520966; doi:10.1186/s40900-020-00233-2)
Supplement: Supplementary file 1 — Additional file 1. [file 40900_2020_233_MOESM1_ESM.pdf]

Thank you for participating in the CONNECT initiative!

DATE: ________________________

Developed under the Canada's Strategy for Patient-Oriented Research (POR), a national initiative promoting patient engagement in health research, the CONNECT project (CONtiNuity of carE and support for autistiC adulTs) represents several milestones for the MSSU and Atlantic Canada. CONNECT is MSSU's first Maritime-based research collaboration between Autistics, caregivers of people living with autism, researchers, service providers, and policy decision-makers on adult autism service needs.

The goal of Patient-Oriented Research is to increase the relevance of research for the patients by fostering their input throughout the research process, including at several key junctures such as the definition and drafting of questionnaire items, the strategizing for participant recruitment, the planning and execution of a knowledge transfer strategy, the interpretation of collected data, the preparation of materials for public release, and the dissemination of study results. The term Patient Partners might feel inappropriate in the context of the CONNECT project and refers here to **autistic adults involved in the project.**

We would like to hear about your participation in the CONNECT initiative in order to learn what worked and what didn't work in this collaboration. The results will be anonymous and used to evaluate and improve our work within MSSU. Ultimately, this evaluation will help draft recommendations on best practices in patient engagement in research to increase the involvement of autistic adults in projects that meet their needs.

1. **What best describes your role in the CONNECT project ? (advisor, leader, ambassador of the CONNECT's results, etc.)**

   ____

2. **Who first approached you about the project?**

   ____

3. Please consider the statements below and answer yes or no next to the question.

I was involved at the beginning of the project during the definition and the drafting of the Maritime Autistic Adults Needs Assessment Survey.	
I was involved in the strategizing for participant recruitment during dissemination of the survey	
I was involved **during and through** the **Autistic Adults Summit**.	
I was involved in the planning and execution of a knowledge transfer strategy (Summit, planning of dissemination of results, etc.)	
I was involved in the interpretation of collected data	
I was involved in the preparation of materials for public release	
I was or will be involved in the dissemination of study results	
Other: Please describe when you got involved in the CONNECT project if not listed in the above.	

QUESTIONS 4 TO 12: Please note that you are not required to limit your answers to the space provided below each question. If you want to provide a longer answer, feel free to add more lines. We are very interested in hearing what you have to say!

4. How did you hear about the project?

____

____

____

____

5. Do you feel that the nature and scope of your engagement was well defined at the beginning of your involvement? Please specify:

____

____

____

____

6. Do you feel that your engagement allowed you an equal opportunity to provide input during the project?

____

____

____

____

7. Do you feel that the team meetings provided you with enough time and information to take part in the discussions? If not, what did the team do to fix the situation?

____

____

____

____

8. How valuable do you feel your engagement was for the CONNECT project?

____

____

____

____

9. How valuable do you feel your engagement was for your personal life?
   What are the lasting impacts of your involvement?

____

____

____

____

10. Do you feel that you have been involved enough?
    Would you have liked to be more engaged? If so, how?

____

____

____

____

11. What were your expectations at the very beginning of this project? Were your expectations met?

____

____

____

____

12. Would you participate again as a patient partner in a similar project if the opportunity arises? If not, why?

____

____

____

____

13. Please provide any other comments you would like to share about the Connect project.

____

____

____

____

Thank you very much for your feedback!
