# Supplementary material for: “We are the stakeholders with the most at stake”: scientific and autism community co-researchers reflect on their collaborative experience in the CONNECT project
Source: Res Involv Engagem. 2020 Sep 27;6:58. doi: 10.1186/s40900-020-00233-2 (PMC7520966; doi:10.1186/s40900-020-00233-2)
Supplement: Supplementary file 2 — Additional file 2. [file 40900_2020_233_MOESM2_ESM.pdf]

CONNECT Project: Feedback Questionnaire for Researchers

Date: ______________________________

1. What best describes your role in the CONNECT project? (for example: advisor, leader, assistant, etc.)

____

2. Before this project, were you familiar with Patient Engagement in research?

☐ Yes ☐ No

Note: If you answered "yes" to the question above, please proceed to questions 2.1 and 2.2.

2.1. Was it practical experience? ☐ Yes ☐ No

2.1.1 How much experience did you have: ☐ A lot ☐ Somewhat ☐ A little

2.2. Was it theoretical knowledge? ☐ Yes ☐ No

2.2.1. How much knowledge did you have: ☐ A lot ☐ Somewhat ☐ A little

3. Do you feel that the nature and scope of Patient Engagement were well defined:

3.1. At the beginning of the project? Please specify:

____
____
____
____

3.2. At the end of the project? Please specify:

____
____
____
____

CONNECT Project: Feedback Questionnaire for Researchers
(*continued*)

4. What were your expectations at the very beginning of this project? Were your expectations met?

5. Overall, how valuable do you feel Patient Engagement was for the CONNECT project?

6. Thinking about your experience in the CONNECT project, what would you improve or change afterwards, if you could?

7. Would you participate again as a researcher in a similar project if the opportunity arises? If not, why?

CONNECT Project: Feedback Questionnaire for Researchers
(*continued*)

8. Please provide any other comments that you would like to share about the CONNECT project.

____
____
____
____
____
